# Population pharmacokinetic analysis of enrofloxacin and its active metabolite ciprofloxacin after intravenous injection to cats with reduced kidney function

**DOI:** 10.1111/jvim.16866

**Published:** 2023-09-20

**Authors:** Jonathan D. Foster, Mahmoud Abouraya, Mark G. Papich, Nancy A. Muma

**Affiliations:** ^1^ Friendship Hospital for Animals Washington DC USA; ^2^ Food and Drug Administration Silver Spring Maryland USA; ^3^ College of Veterinary Medicine North Carolina State University Raleigh North Carolina USA; ^4^ Department of Pharmacology and Toxicology University of Kansas Lawrence Kansas USA

**Keywords:** drug prescribing, feline, fluroquinolone, renal failure

## Abstract

**Background:**

It is unknown if enrofloxacin accumulates in plasma of cats with reduced kidney function.

**Hypothesis:**

To determine if enrofloxacin and its active metabolite ciprofloxacin have reduced clearance in azotemic cats.

**Animals:**

Thirty‐four cats hospitalized for clinical illness with variable degree of kidney function.

**Methods:**

Prospective study. After enrofloxacin (dose 5 mg/kg) administration to cats, sparse blood sampling was used to obtain 2 compartment population pharmacokinetic results using nonlinear mixed‐effects modeling. Plasma enrofloxacin and ciprofloxacin concentrations were measured and summed to obtain the total fluoroquinolone concentration. A model of ciprofloxacin metabolism from enrofloxacin was created and evaluated for covariate effects on clearance, volume of distribution, and the metabolic rate of ciprofloxacin generation from enrofloxacin.

**Results:**

Body weight was the only covariate found to affect total fluoroquinolone volume of distribution (effect 1.63, SE 0.19, *P* < .01) and clearance (effect 1.63, SE 0.27, *P* < .01). Kidney function did not have a significant effect on total fluoroquinolone clearance (median 440.8 mL/kg/h (range 191.4‐538.0 mL/kg/h) in cats with normal kidney function, 365.8 mL/kg/h (range 89.49‐1092.0 mL/kg/h) in cats with moderate kidney dysfunction, and 308.5 mL/kg/h (range 140.20‐480.0 mL/kg/h) in cats with severe kidney dysfunction (*P* = .64). Blood urea nitrogen concentration influenced the metabolic generation of ciprofloxacin from enrofloxacin (effect 0.51, SE 0.08, *P* < .01), but other markers of kidney function did not.

**Conclusions and clinical importance.:**

Adjustment of enrofloxacin dosage is not indicated for azotemic cats.

**Abbreviations**
AIC: Akaike information criterion
AUC: area under concentration curve
BUN: blood urea nitrogen
CKD: chronic kidney disease
*C*_max_
: peak serum or plasma drug concentration
CV: coefficient of variability
GFR: glomerular filtration rate
IV: intravenous
NLME: nonlinear mixed‐effects modeling
SDMA: symmetric dimethylarginine
*V*_d_
: volume of distribution

## 1 INTRODUCTION

Treatment of bacterial pyelonephritis requires an adequate concentration of antibiotic at the site of infection within the renal parenchyma.^1^, ^2^, ^3^, ^4^ Fluoroquinolone antibiotics are recommended as the empiric therapy for pyelonephritis when culture results are pending^5^ as there is epidemiologic data that fluoroquinolone antibiotics have favorable antibacterial activity among urinary tract pathogens in dogs and cats.^6^, ^7^, ^8^, ^9^ The choice of antibiotic should be amended according to aerobic urine culture antimicrobial susceptibility testing.

In the United States, injectable enrofloxacin is only approved for use in dogs, but is frequently used extra‐label in cats for susceptible bacterial infections as it is the only injectable fluoroquinolone available in this country. Enrofloxacin is extensively metabolized by the liver, being N‐dealkylated to form ciprofloxacin, the main active metabolite. The antibacterial effects of enrofloxacin and ciprofloxacin are additive.^10^

After oral administration of enrofloxacin to cats, the majority of the drug accumulated within urine, suggesting renal excretion as the primary means of elimination.^11^ The exact means of renal elimination is unknown, but is likely because of a combination of glomerular filtration of unbound drug and active tubular secretion.^12^

Patients with kidney dysfunction might have alterations in renal drug clearance. If glomerulotubular balance is intact, a reduction in glomerular filtration rate (GFR) will be accompanied by proportional reductions in tubular secretion and absorption. The net renal drug elimination is determined by filtration and tubular modification. Kidney disease accompanied by reduced GFR would result in decreased filtration of the unbound forms of these drugs. Similarly, reduced renal tubular secretory capability would decrease renal clearance of ciprofloxacin and perhaps enrofloxacin.^13^, ^14^

Retinal degeneration occurs in some cats receiving high dosages (>8 mg/kg/day) of enrofloxacin.^15^ It is unclear if retinal injury is related to peak drug concentration or cumulative drug exposure. Further analysis of the risk of retinal injury suggested cat age and dose of enrofloxacin might have contributory roles.^16^ Older cats might be more likely to have kidney disease, which could result in greater exposure to enrofloxacin and ciprofloxacin concentrations and higher risk of developing retinal degeneration.

Other fluoroquinolones have been studied in humans and animals with decreased kidney function. Ciprofloxacin, enrofloxacin, and marbofloxacin have reduced clearance and increased drug exposure in individuals with reduced kidney function.^17^, ^18^, ^19^, ^20^, ^21^ The dosage or administration frequency of fluoroquinolones might need to be adjusted in patients with kidney dysfunction to prevent excessive drug accumulation. Although research has not been performed, some authors recommend extending the dosing interval of fluoroquinolone antibiotics to dogs and cats with kidney dysfunction.^22^

The pharmacokinetics of intravenously administered enrofloxacin is reported for healthy adult cats and kittens,^23^ but there have been no evaluations in cats with either experimental or naturally occurring kidney disease.

The purpose of this study is to determine if enrofloxacin and its active metabolite ciprofloxacin have reduced clearance in azotemic cats.

## 2 MATERIALS AND METHODS

This was a prospective population pharmacokinetic study utilizing nonlinear mixed‐effects modeling (NLME). All cats were enrolled and treated at [masked for review], after informed consent by their owners from January 1, 2019 through September 1, 2021.

Client‐owned cats that were administered enrofloxacin IV at the discretion of their primary veterinarian for suspected bacterial infection were eligible for enrollment. Cats that had received any fluoroquinolone in the previous 7 days were ineligible for enrollment in this study. Additional exclusion criteria were cats with preexisting retinal lesions or blindness, severe concurrent disease including anemia (hematocrit <20%), hypoxia, congestive heart failure, hypotension, and hypovolemia. Fractious cats that would experience excessive stress during venipuncture were ineligible for enrollment.

All cats received 5 mg/kg IV injection of enrofloxacin (Baytril 2.27% solution, Elanco, Greenfield, IN). The volume of enrofloxacin was diluted 1:1 with sterile saline and this final solution was administered through a peripheral IV catheter over 30 minutes. No other concurrent drug or fluid was administered through that venous catheter during drug administration. Samples were collected after the complete volume of enrofloxacin had been administered. Although sampling was only performed in the 24 hours after the initial IV dose of enrofloxacin, all cats continued to receive antibiotic therapy as clinically indicated; transition to another antibiotic based on culture and antimicrobial susceptibility report was performed as indicated.

The cats enrolled in this study were hospitalized for the management of their illness and could not tolerate intensive sampling for individual pharmacokinetic analysis. Instead, a population‐based approach to modeling the pharmacokinetics was designed using NLME evaluation, that would allow for fewer samples to be obtained from each cat. Sparse sampling was performed so that each cat was scheduled to have only 3 blood samples obtained in the 24 hours after enrofloxacin administration. The sample schedule was designed to optimize time points according to previously published pharmacokinetic variables in healthy cats.^23^ Cats were randomly assigned to a specific sparse sampling schedule (see Table S1).

At each sampling time point, 1.5 mL of whole blood was collected via venipuncture into heparinized tubes, centrifuged, and plasma separated and stored at −80°C. Stored samples were shipped to North Carolina State University College of Veterinary Medicine for analysis. Concentrations of enrofloxacin and ciprofloxacin were determined using high‐pressure liquid chromatography using a validated assay.^23^

The antibacterial effects of enrofloxacin and ciprofloxacin are additive.^10^, ^24^ Therefore, the total fluroquinolone concentration (sum of enrofloxacin and ciprofloxacin concentrations) was used for pharmacokinetic analysis.

As plasma ciprofloxacin concentration is also dependent on the rate of metabolism of enrofloxacin into ciprofloxacin, secondary modeling was performed to determine the relationship between formation of ciprofloxacin and clearance of both ciprofloxacin and enrofloxacin. Modeling was used to identify inter‐ and intra‐subject variability and covariates that significantly affect the pharmacokinetics of the drug.

The models were parameterized by drug clearance. Final model selection was based on goodness of fit plots, statistical significance between models using twice the negative log likelihood (−2LL), Akaike information criterion (AIC),^25^ a goodness of fit measure based on the log likelihood adjusted for the number of variables and degrees of freedom in the model, obtained in Phoenix NLME, and coefficient of variation of variable estimates. The model with the smallest AIC was selected as the final model.

Modeling population pharmacokinetics yields better estimates of intersubject variability than traditional approaches, while allowing the individual to be accounted for in modeling variability.^26^ Naturally occurring kidney disease in people and experimentally induced kidney dysfunction in rats causes between 32.5% and 73.4% decrease in clearance of enrofloxacin or ciprofloxacin.^17^, ^18^, ^19^, ^20^ Based on enrofloxacin pharmacokinetics in healthy adult cats (mean clearance 257 ± 54 mL/kg/h),^23^ 4 normal cats and 4 with decreased kidney function (characterized by a serum creatinine concentration >2.4 mg/dL) need to be enrolled to have 80% power to detect a 32.5% reduction in drug clearance. Because enrolled cats will have variable renal function, ranging from normal to markedly reduced, a minimum of 24 cats was targeted for enrollment to allow for 8 cats with normal, moderately decreased, and markedly decreased kidney function to be included in analysis, as suggested by regulatory agencies, and exceeding the a priori sample size determination.^27^

Through sparse‐sampling, initial pharmacokinetic variables of plasma total fluoroquinolone concentration (sum of plasma enrofloxacin and ciprofloxacin concentration at a specific time point) were estimated using naive pooled modeling and noncompartmental methods. These estimates were analyzed with NLME population pharmacokinetic modeling with commercial software (Phoenix NLME software, Certara, Princeton, NJ). Once the final model was obtained for the population, an examination of covariates was performed to determine additional factors that might explain the variability in clearance of enrofloxacin and ciprofloxacin.

Interindividual (between‐subject) variability (variance of a variable among different subjects) was expressed using an exponential error model according to the equation:

$$P_{i}=P_{\mathrm{pop}}\times \exp^{\left(\mathrm{\eta iP}\right)}$$

where *P* is the variable of interest for the individual *i*, *P*_pop_ is *θ* (theta), the typical value for the population estimate of the variable of interest, and *ηiP* is the *η* (eta) for the individual and variable of interest. The *η* values were assumed to be independent and have a normal distribution with a mean of 0 and variance of *ω* ^2^. A multiplicative model was chosen (among additive, log‐additive, power, and mixed error models) to describe the residual** random variability (*ε*) of the data, where *ε* is the residual intrasubject (within subject) variability with a mean of 0 and a variance of *σ* ^2^, according to the equation:

$${\text{Cobs}}_{\mathrm{ij}}={\text{Cpred}}_{\mathrm{ij}}\times \left(1+\varepsilon_{\mathrm{ij}}\right)$$

where Cobs_*ij*_ is the observed concentration for subject *i* at time *j* for the individual and Cpred_*ij*_ is the model predicted concentration for subject *i* at time *j* plus the error value (*ε*_*ij*_) adjustment for subject *i* at time *t*_*j*_ (multiplicative residual error).

The contribution of covariate to clearance and volume of both compartments was evaluated after the population model was built. Sex was added to the base model as a categorical covariate (where female = 0 and male = 1). Covariates including cat age and weight as well as biochemical variables including serum creatinine, symmetric dimethylarginine (SDMA), and blood urea nitrogen (BUN) concentrations were evaluated as continuous variables. The effect of each covariate on the volume and clearance was evaluated using a likelihood ratio test, and a *P* value <.01 was significant for adding a covariate and a *P* value <.001 was significant for the removal of a covariate. The final model was validated using the bootstrap technique.

Internal model validation was performed via bootstrap modeling with 300 replicates.

Continuous data was analyzed for normality via the D'Agostino Pearson test using a commercial statistical program (Graphpad Prism 9.3). Normally distributed data are reported as mean and SD, data that are not normally distributed data are reported as median and range. Pharmacokinetic variables were compared between groups of cats stratified by renal function (normal kidney function: serum creatinine concentration ≤2.4 mg/dL [within the reference interval], moderate kidney dysfunction: serum creatinine concentration 2.5‐10 mg/dL, and severe kidney dysfunction: serum creatinine concentration >10 mg/dL) via a Kruskal‐Wallis test. A *P* value <.05 was considered significant.

## 3 RESULTS

A total of 34 cats were enrolled in this study, 18 were spayed females and 16 were castrated males. The mean age was 11.4 ± 4.3 years, and median weight was 3.8 kg (range 1.8‐8.8 kg). The mean serum creatinine concentration and SD of the mean were 7.5 ± 6.0 mg/dL, BUN 137.6 ± 103.0 mg/dL, and SDMA 39.8 ± 27.0 μg/dL. Nine cats had normal kidney function (median serum creatinine concentration 1.6, range 0.9‐2.2 mg/dL), 15 had moderate kidney dysfunction (median serum creatinine concentration 5.6, range 2.7‐8.6 mg/dL), and 10 had severe kidney dysfunction (median serum creatinine concentration 13.3, range 11.9‐21.5 mg/dL). All cats received 5 mg/kg enrofloxacin IV, with a mean total dose of 20.5 ± 7.4 mg. Three cats were enrolled in sampling schedule 1, 10 in schedule 2, 4 in schedule 3, 1 in schedule 4, 6 in schedule 5, 4 in schedule 6, and 6 in schedule 7 (see Table S1).

### 3.1 Noncompartmental analysis

**FIGURE 1:**
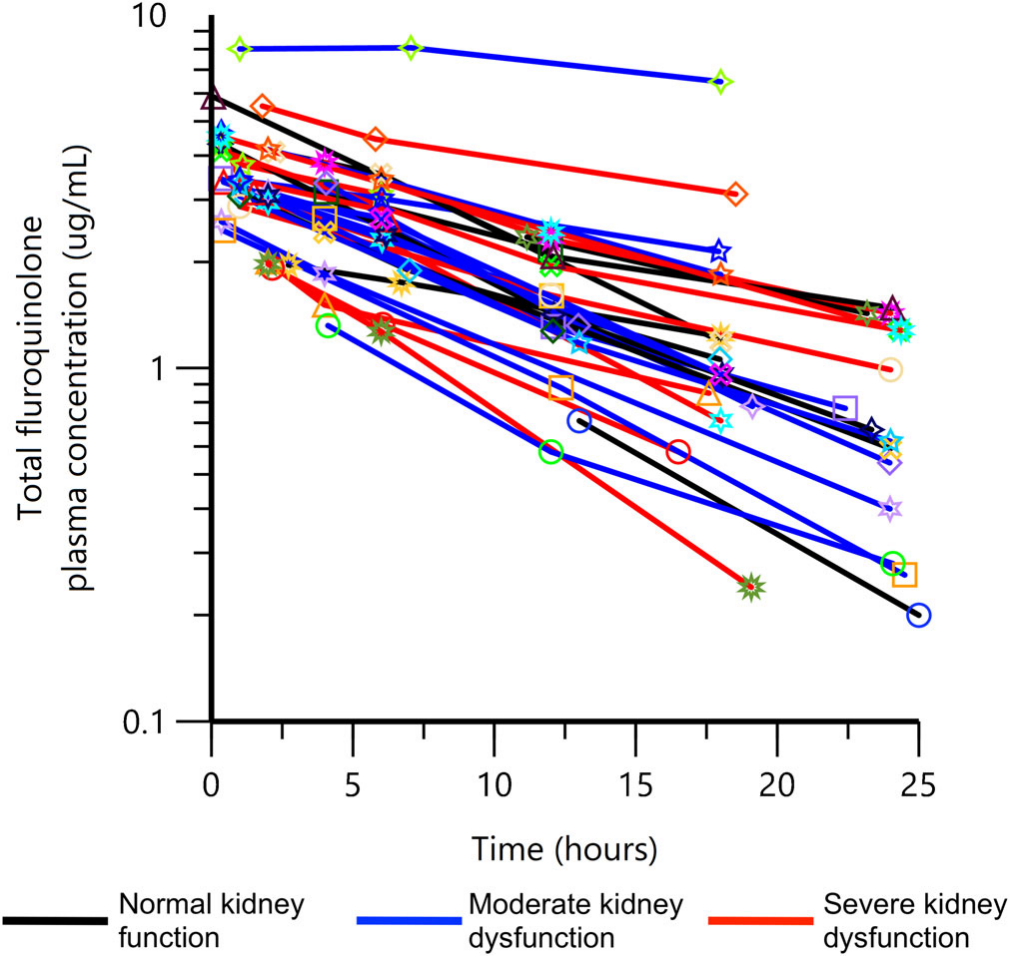
Semi‐logarithmic spaghetti plots of total fluroquinolone plasma concentrations over 24 hours after a single IV administration (nominal dose of 5 mg/kg) of enrofloxacin in 34 cats.

Exactly 98 samples were obtained from the 34 enrolled cats. Four cats only had 2 blood samples analyzed, as each of these cats had a single blood sample that was lost in processing. Noncompartmental pharmacokinetic analysis was performed and is shown in Figure 1 as total fluroquinolone concentration vs time.

There was no difference in the observed area under the curve through 24 hours (AUC_0→24_) after dose administration between groups with different renal function (*P* = .66); 41.44 μg h/mL (range 28.29‐69.29 μg h/mL) in cats with normal kidney function, 39.79 μg h/mL (range 18.66‐136.3 μg h/mL) in cats with moderate kidney dysfunction, and 34.95 μg h/mL (range 19.95‐78.5 μg h/mL) in cats with severe kidney dysfunction. There was no difference in observed clearance between groups with different renal function (*P* = .64); 440.8 mL/kg/h (range 191.4‐538.0 mL/kg/h) in cats with normal kidney function, 365.8 mL/kg/h (range 89.49‐1092.0 mL/kg/h) in cats with moderate kidney dysfunction, and 308.5 mL/kg/h (range 140.20‐480.0 mL/kg/h) in cats with severe kidney dysfunction.

The data was best modeled by 2‐compartment kinetics demonstrated goodness of fit evaluation and by the lower mean AIC −23.61 compared with −8.97 for 1‐compartment model.

A 2‐compartment model was created and evaluated using additive, multiplicative, and additive with multiplicative error models, which found a multiplicative model to have the best performance (AIC 207.14 vs 209.61 for the proportional model and 230.24 for the additive model).

### 3.2 Covariate model

**FIGURE 2:**
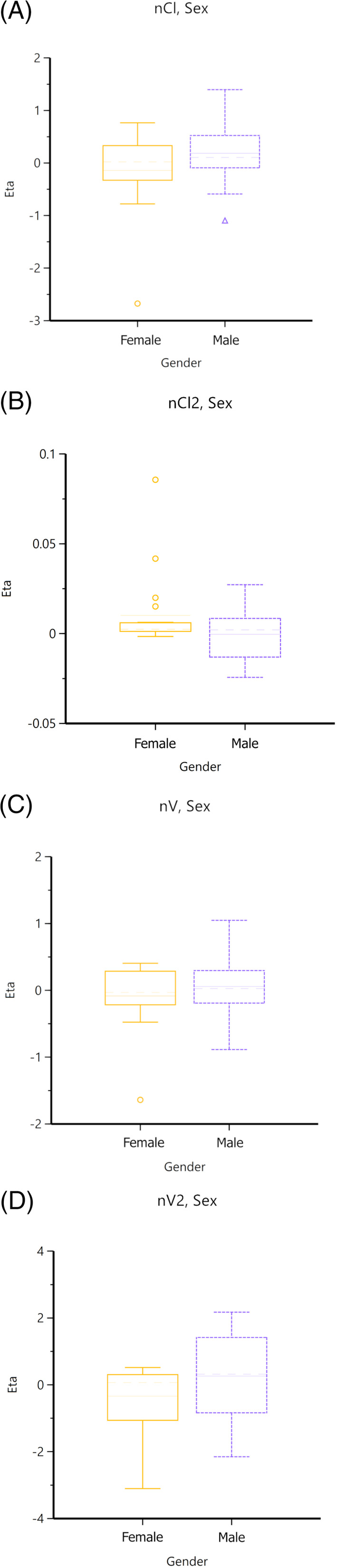
Box plots of effect of sex on clearance and volume of both compartments.

**FIGURE 3:**
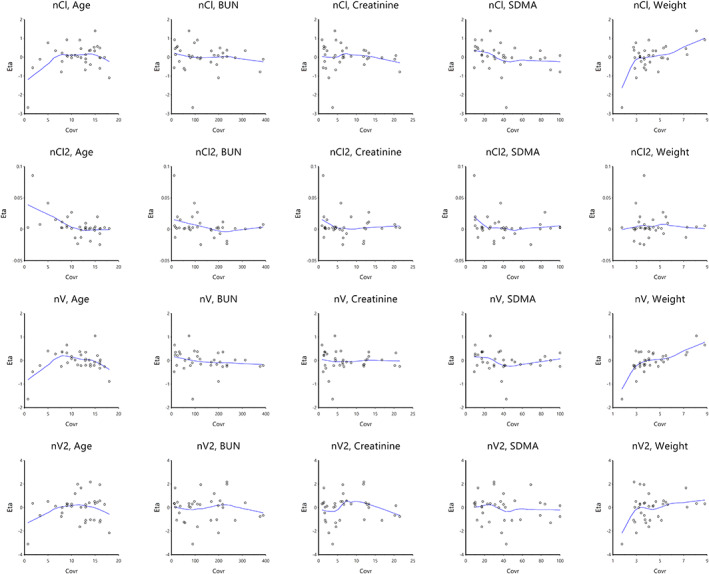
Population plots of the effect of each continuous covariate on clearance and volume of both compartments. Body weight significantly contributed to both clearance and volume of distribution (*P* < .01).

Using the 2‐compartment model with multiplicative error regression, a stepwise search for covariates was performed (Figures 2 and 3). Forward addition and backward deletion iteration were performed. Only body weight was found to significantly affect the volume and clearance of the first compartment. All other covariates were found to not significantly affect volume or clearance of either compartment.

The final population pharmacokinetic model variables are shown in Table 1. In this model body weight was centered around the mean population body weight, 3.8 kg. The contribution of ciprofloxacin to the total fluoroquinolone concentration is reported as the ciprofloxacin: quinolone ratio, essentially ciprofloxacin's percentage of total fluoroquinolone concentration at any time point.

**TABLE 1:** Enrofloxacin population pharmacokinetic variables in cats.

Parameter	Estimate	SE	CV%
*θ V*_d_1 (mL/kg)	4794.7	359.6	7.22
*V*_d_2 (mL/kg)	2.9 × 10^−4^	5.1 × 10^−5^	17.6
*θ* Cl1 (mL/kg/h)	254.3	31.8	12.49
Cl2 (mL/kg/h)	132.1	27.4	20.71
d*V*_d_1weight	1.6	0.2	13.75
dCl1weight	1.6	0.3	16.38
AUC_0→24_ (μg h/mL)	46.0	3.1	36.33
Ciprofloxacin: quinolone ratio	0.18	0.02	86.39

*Note*: *θ V*_d_1 is the theta (typical value) for quinolone volume of distribution of the first compartment; *V*_d_2 is the volume of distribution of quinolone within the second compartment; *θ* Cl1 is the theta for quinolone clearance from the first compartment; Cl2 is the clearance of quinolone from the second compartment; d*V*_d_1weight is the effect of the covariate weight on the volume of distribution of the first compartment; dCl1weight is the effect of the covariate weight on the quinolone clearance of the first compartment; AUC_0→24_ is area under the curve for the concentration versus time profile from dosing (time 0 hour) through 24 hours post‐dosing; Ciprofloxacin:quinolone is the ciprofloxacin‐to‐total quinolone (enrofloxacin plus ciprofloxacin) plasma concentration ratio.

**FIGURE 4:**
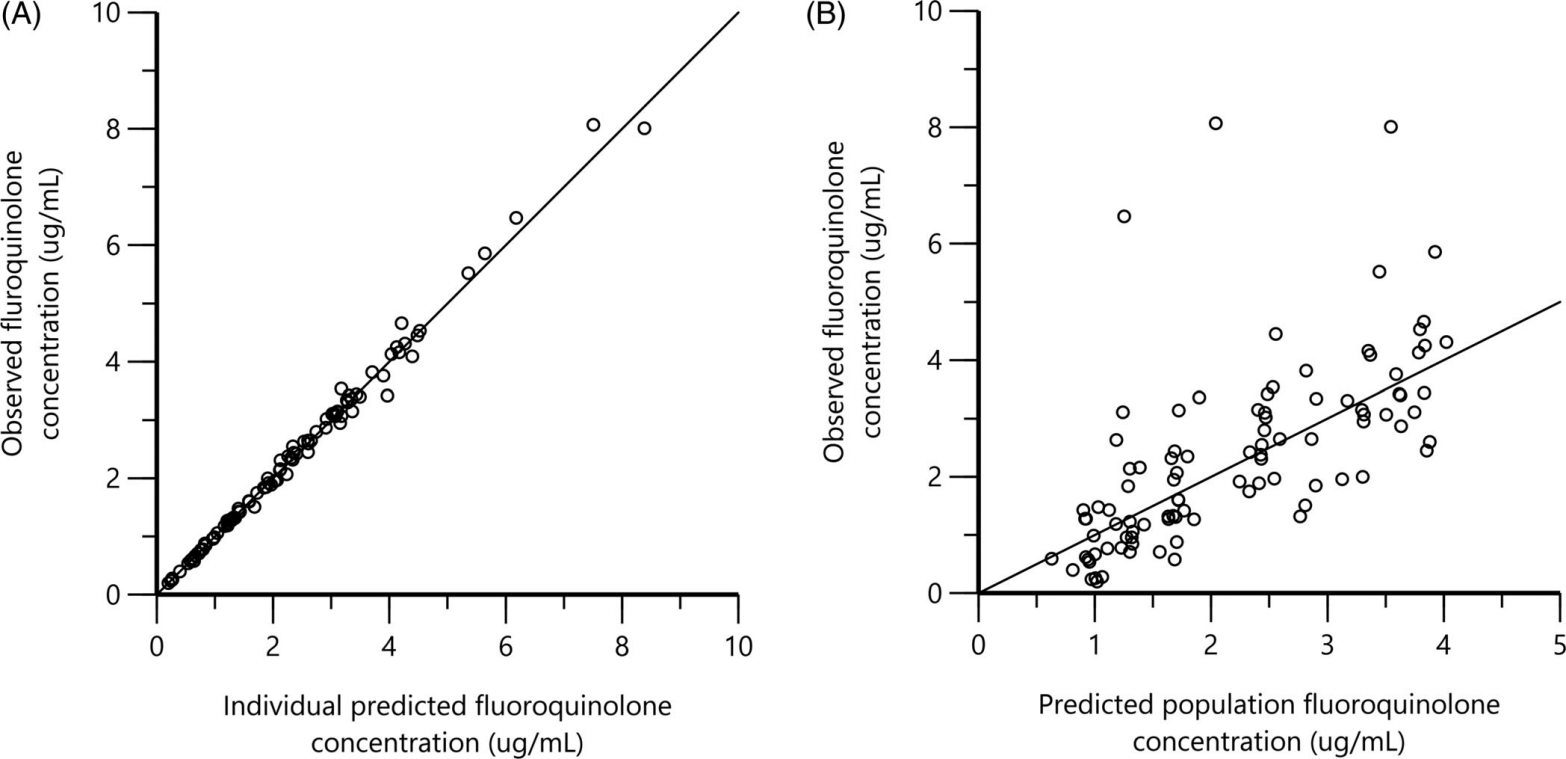
Predicted vs observed plasma fluoroquinolone concentrations from the nonlinear mixed‐effects model.

The basic goodness‐of‐fit diagnostic plots for the final population pharmacokinetic model are shown in Figure 4. Individual fluoroquinolone concentrations are predicted well by the model (Figure 4A), with data evenly distributed about the line of identity, indicating an appropriate structural model could be found for most individuals. There is no major indication for bias in the population component of the model (Figure 4B) as data remains evenly distributed about the line of identity.

### 3.3 Model validation

Model validation was performed via bootstrap simulation with 300 replicates. The results of the bootstrap simulation are presented alongside model estimates in Table S2. Bootstrap replicate observations were similar to model estimates for all variables except the fluoroquinolone volume of distribution of the second compartment, which was found to be higher and have a greater coefficient of variability in the bootstrap simulation. This suggests individual variability in this variable might not be completely accounted for by the model.

From the bootstrap simulations, a predictive check was performed using observed and simulated predicted plasma fluoroquinolone concentration (Figure S1). There was good agreement between observed and predicted data, but some kurtosis was observed in the predicted quantiles beyond 12 hours after dosing.

### 3.4 Modeling of enrofloxacin metabolism to ciprofloxacin

A graphical model was created to investigate the conversion of enrofloxacin to ciprofloxacin as well as their respective clearances (Figure S2). The concentration of enrofloxacin and ciprofloxacin was evaluated according to the relationship illustrated by this model to determine the clearance of enrofloxacin to ciprofloxacin (also considered the metabolism of enrofloxacin to ciprofloxacin) and the clearance of both compounds into urine. Results of this model are shown in Table 2. Estimates of the *V*_d_ of enrofloxacin and its clearance had little variability within the study population. The *V*_d_ of ciprofloxacin, its clearance, as well as the metabolism of enrofloxacin to ciprofloxacin had large coefficient of variability. A stepwise search for covariates was performed to identify factors that might account for this variability.

**TABLE 2:** Results from modeling enrofloxacin and its metabolite ciprofloxacin.

Parameter	Estimate	SE	CV%
*V*_d_ enrofloxacin (mL/kg)	5797.5	323.5	5.4
Clearance of enrofloxacin into urine (mL/kg/h)	499.1	42.6	8.5
*V*_d_ ciprofloxacin (mL/kg)	22.7	4.2	18.7
Clearance of ciprofloxacin into urine (mL/kg/h)	4.6	0.8	16.9
Metabolism of enrofloxacin to ciprofloxacin (mL/kg/h)	0.13	0.03	22.9

Abbreviation: *V*_d_, volume of distribution.

**FIGURE 5:**
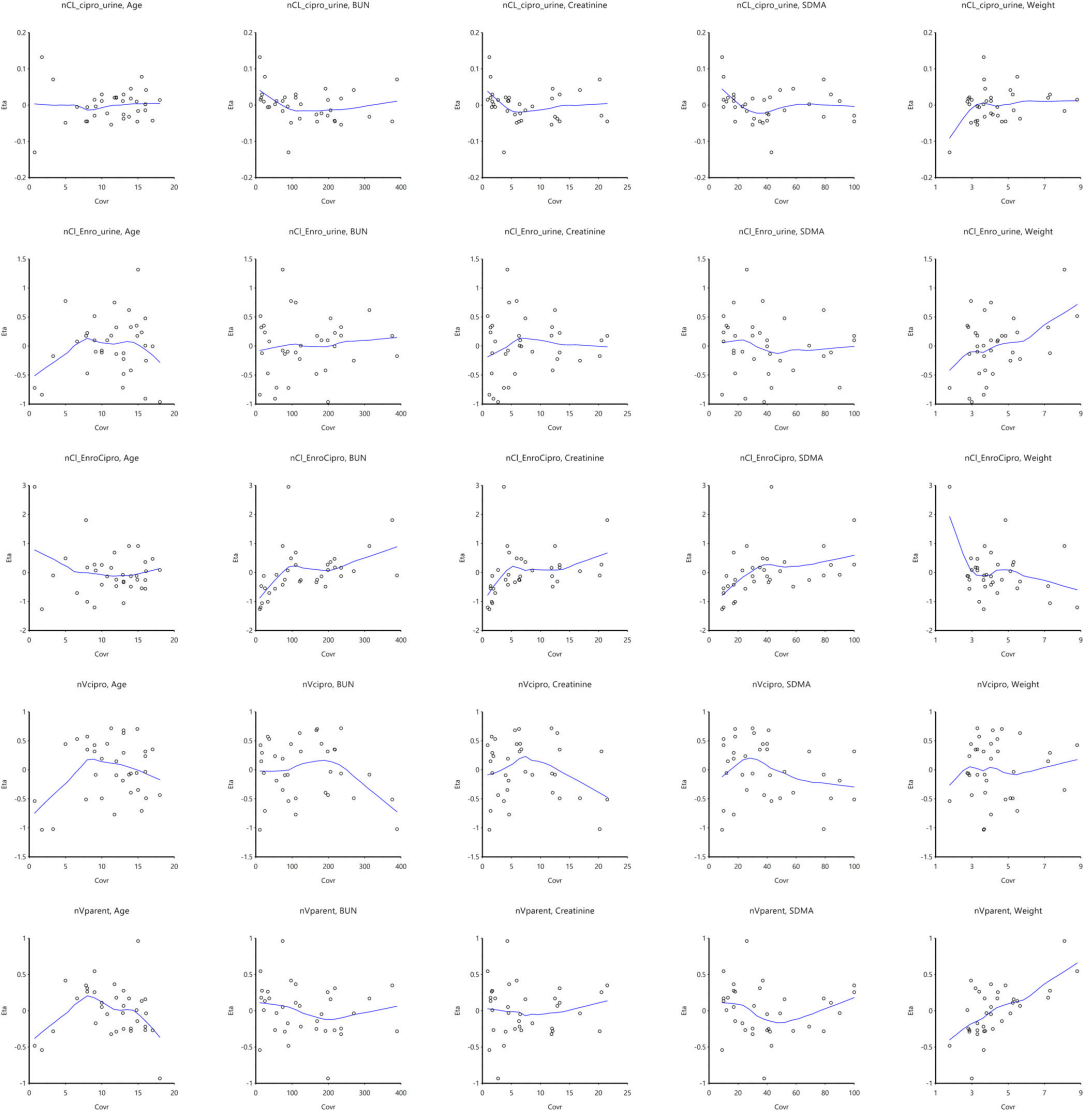
Population plots of the effect of each continuous covariate on kinetics of enrofloxacin and metabolite ciprofloxacin.

A stepwise covariate search identified BUN as a covariate that affected the metabolism of enrofloxacin to ciprofloxacin and body weight as a covariate that affected *V*_d_ of enrofloxacin (Figure 5). The final population variables of the metabolite model are shown in Table 3.

**TABLE 3:** Final covariate population model of enrofloxacin and ciprofloxacin kinetics.

Parameter	Estimate	SE	CV%
*θ V*_d_ Enrofloxacin (mL/kg)	5797.5	313.5	5.4
Clearance of enrofloxacin into urine (mL/kg/h)	499.1	42.6	8.5
*V*_d_ ciprofloxacin (mL/kg)	22.6	4.2	18.7
Clearance of ciprofloxacin into urine (mL/kg/h)	4.6	0.8	16.9
*θ* Metabolism of enrofloxacin to ciprofloxacin (mL/kg/h)	0.1	0.03	22.9
d*V*_d_ Enrofloxacin‐Weight	0.7	0.15	20.0
dCl Enrofloxacin metabolism to ciprofloxacin BUN	0.5	0.08	15.5

*Note*: *θ V*_d_ enrofloxacin is the theta (typical value) for enrofloxacin's volume of distribution *V*_d_ ciprofloxacin is the volume of distribution of ciprofloxacin; *θ* Cl1 is the theta for quinolone clearance from the first compartment; *θ* metabolism is the theta of clearance of enrofloxacin to ciprofloxacin (representing the formulation of the active metabolite ciprofloxacin from enrofloxacin); dCl Enrofloxacin is the effect of the covariate BUN on the metabolic clearance of enrofloxacin to ciprofloxacin; d*V*_d_1weight is the effect of the covariate weight on the volume of distribution of enrofloxacin.

## 4 DISCUSSION

Client‐owned cats in a hospital cannot be sampled as frequently as research cats. Therefore, we used a population pharmacokinetic approach with a sparse sampling design. Sparse sampling of cats with naturally occurring disease receiving IV enrofloxacin allowed for population pharmacokinetic modeling via a NLME approach. This method investigated interactions of individual variability on pharmacokinetic variables. Kidney function assessed by serum creatinine, BUN, and SDMA concentrations did not contribute significantly to the population variability (between‐subject variability) for total fluoroquinolone clearance or volume of distribution. While population modeling allowed for renal function to be assessed as a continuous variable via these variables, when cats were grouped according to the magnitude of kidney dysfunction, there was no difference observed in AUC_0→24_ or clearance among groups. Because volume of distribution and clearance are derived from the AUC it is not unexpected that we did not see significant differences in these variables. Only body weight affected the volume of the first compartment as well as its clearance. Further evaluation of enrofloxacin pharmacokinetics in cats with larger body weight (>7 kg) might be indicated based upon this finding.

Comparing the results of this study to earlier studies of enrofloxacin pharmacokinetics in healthy adult cats,^23^, ^28^ a similar ciprofloxacin: total fluoroquinolone ratio was observed; however, the current population had a larger *V*_d_, clearance, and AUC_0→24_ of the total fluroquinolone concentration. The cause for these differences is undetermined but could be because these cats were all hospitalized for management of illness rather than healthy research adults. Intravenous fluid therapy was given to all cats according to their hydration requirements according to the discretion of the primary veterinarian. Fluid therapy might increase the *V*_d_ of enrofloxacin and ciprofloxacin because of extracellular volume expansion, but this relationship has not been tested.

Bootstrap validation of the model showed a high CV for the *V*_d_ and total fluoroquinolone clearance of the second compartment, as was predicted by the model. This compartment represents extravascular sites of fluoroquinolone accumulation such as within tissues. The cause for the variability observed here is unknown and might be reflective of drug accumulation at the site of infection, which might vary according to the severity of disease. The *V*_d_ and clearance of this second compartment were not affected by the presence of renal disease or any other covariates.

This study modeled the total fluoroquinolone concentration in plasma. Ciprofloxacin is an active metabolite of enrofloxacin, and both exhibit antibiotic effects through inhibition of topoisomerase IV.^24^, ^29^ A model of ciprofloxacin generation from enrofloxacin was created so that individual enrofloxacin and ciprofloxacin clearance, *V*_d,_ and the rate of metabolism of enrofloxacin to ciprofloxacin could be investigated. Covariate analysis identified the cat's BUN to be a covariate affecting the metabolism of enrofloxacin to ciprofloxacin. As BUN increased, the rate of this metabolism also increased. This suggests cats that are more azotemic have higher rates of ciprofloxacin formation, however serum creatinine and SDMA concentrations were not found to be significant covariates on this metabolic rate. Serum creatinine and SDMA concentrations are reasonable biomarkers for GFR in cats,^30^, ^31^ however BUN has poor specificity in detecting decreased GFR.^32^ Serum creatinine predicts GFR better than BUN in cats.^33^ The poor utility of BUN as a biomarker of GFR combined with the lack of influence of serum creatinine and SDMA concentration on the metabolism of ciprofloxacin from enrofloxacin undermine the role of kidney disease in enrofloxacin metabolism. It is unknown why BUN and no other biomarkers of GFR would be associated with this metabolism, however it is unlikely that GFR affects the metabolism as BUN, serum creatinine, and SDMA would be expected to be significant covariates within the model. Enrofloxacin is N‐dealkylated within the liver to form ciprofloxacin and there is no clear connection between GFR and this metabolism. Anephric humans who lack any GFR still have substantial elimination and clearance of ciprofloxacin, suggesting nonrenal pathways might be utilized when renal elimination is reduced.^34^ Ciprofloxacin dosing recommendations in people are contradictory, with some authors suggesting no change be made^35^ and others recommending dose reduction only in patients with severely reduced GFR.^34^, ^36^ It us unknown if the metabolism of ciprofloxacin from enrofloxacin is the rate limiting step in cats, and possibly unaffected by kidney function. Based on the current study, no change in dosage or administration schedule of enrofloxacin is indicated in cats with kidney disease.

Studies in other species show decreased clearance of fluoroquinolone antibiotics attributed to kidney disease. Experimental models in rats and in humans with kidney disease show plasma accumulation of ciprofloxacin and dose adjustment is required.^17^, ^18^, ^20^ Dogs with experimentally reduced GFR have decreased clearance of N‐oxide‐marbofloxacin, but not the parent drug marbofloxacin.^21^ This suggests that not all fluroquinolones are affected similarly by reductions in GFR. In the absence of data, some have proposed that enrofloxacin dosing interval be prolonged in cats with kidney disease.^22^ Data from the current study does not support this practice. A reduction in the dose, or a longer dose interval could decrease the concentrations needed for adequate eradication of an infection.

Population pharmacokinetic modeling was the appropriate approach for this population through sparse sampling, which allowed for a study to be performed in cats with clinical disease without excessive blood sampling. This technique is well suited for hospitalized cats, where they might not tolerate, or the owner might not consent to dense sampling. Other pharmacokinetic studies have been performed in dogs and cats with naturally occurring kidney disease. Ampicillin and amoxicillin have higher plasma drug concentrations in azotemic dogs and cats compared with healthy controls.^37^, ^38^ However, neither of these studies stratified dogs and cats according to the magnitude of reduction in kidney function. Therefore, it is challenging to accurately predict changes in drug elimination in dogs with mild vs severe kidney disease. The NLME modeling performed in the present study allows for evaluation of the effect of reduced kidney function on pharmacokinetics and can quantify these effects. Such an approach can generate dosing nomograms to more accurately adjust drug dosing or administration interval to obtain drug elimination as seen in animals with normal kidney function. Because kidney disease was not found to affect the clearance of fluoroquinolone, a nomogram based on kidney function is not needed.

This study has several strengths and limitations. Cats were enrolled across the spectrum of reduced kidney function, which produced a larger sample size than others reported for cats in any pharmacokinetic study. Kidney function was not directly assessed through GFR measurement. It is possible that some cats might have a serum creatinine concentration within the population reference range but have a lower than normal in GFR. While no cat had overt hepatic failure clinically, liver function was not specifically tested in these cats as it was not clinically indicated. Hepatic failure in cats is usually clinically apparent, and we did not suspect that these cats had signs consistent with liver failure. However, it is unknown if any cat had decreased hepatic metabolism of enrofloxacin that could affect overall clearance. This study also evaluated drug clearance after the first dose of enrofloxacin. Although we cannot predict what might occur from multiple doses, the pharmacokinetic variables reported here can be adequately assessed from a single dose. Healthy cats have minimal total fluoroquinolone present within serum 24 hours after IV administration.^23^, ^28^ This study found some cats to have higher total fluoroquinolone concentrations at 24 hours compared with this study (see Figure 1), but additional study is required to measure the pharmacokinetics from multiple dosing.

In conclusion, decreased kidney function as evaluated by serum creatinine, SDMA, and BUN concentration did not affect plasma total fluoroquinolone, enrofloxacin, or ciprofloxacin clearance. Adjustment of enrofloxacin dosage is not indicated for azotemic cats, however further study of repeated doses is needed.

## CONFLICT OF INTEREST DECLARATION

Jonathan D Foster is a board member of the International Renal Interest Society and an advisory board member for Elanco. His participation in the Elanco advisory board began after oral presentation of this research and did not overlap when the research was performed. Mahmoud Abouraya is an employee of the Food and Drug Administration (FDA). The information in these materials is not a formal dissemination of information by FDA and does not constitute an advisory opinion, does not necessarily represent the formal position of FDA, and does not bind or otherwise obligate or commit the agency to the views expressed. This collaboration should not be construed as FDA endorsement or preference for a company or specific modeling software. This study was not used for FDA drug approval. Mark G Papich is a current member, and former chair holder of the CLSI‐VAST subcommittee. No other authors declare a conflict of interest.

## OFF‐LABEL ANTIMICROBIAL DECLARATION

Injectable enrofloxacin is not labeled for administration in cats. Oral enrofloxacin is FDA approved for use in cats; injectable enrofloxacin is FDA approved for dogs only.

## INSTITUTIONAL ANIMAL CARE AND USE COMMITTEE (IACUC) OR OTHER APPROVAL DECLARATION

Animal Clinical Investigations IACUC Study Code: Enro PK. ACI LLC, Chevy Chase MD.

## HUMAN ETHICS APPROVAL DECLARATION

Authors declare human ethics approval was not needed for this study.

## Supporting information


**Table S1.** Sparse sampling grid.

**Table S2.** Results of 300 bootstrap simulations and original model estimates.

**Figure S1.** Visual check of predicted and observed total fluoroquinolone concentrations vs time.

**Figure S2.** Graphical model of enrofloxacin metabolism to ciprofloxacin and their respective clearances.
