# Supplementary material for: Population pharmacokinetic analysis of enrofloxacin and its active metabolite ciprofloxacin after intravenous injection to cats with reduced kidney function
Source: J Vet Intern Med. 2023 Sep 20;37(6):2230–40. doi: 10.1111/jvim.16866 (PMC10658592; doi:10.1111/jvim.16866)
Supplement: Supplementary file 1 — Table S1. Sparse sampling grid. [file JVIM-37-2230-s004.pdf]

Supplemental Table 1. Sparse sampling grid. An "X" in the grid indicates a time that a blood sample was obtained following administration of 5 mg/kg enrofloxacin intravenously at time 0 (timer began at the completion of the enrofloxacin infusion).

**Schedule**	**20 min**	**1 hr**	**2 hr**	**4 hr**	**6 hr**	**12 hr**	**18 hr**	**24 hr**
1	X	X			X			
2			X		X		X	
3		X				X		X
4	X			X				X
5	X					X		X
6				X		X		X
7		X			X		X	
