# Supplementary material for: Population pharmacokinetic analysis of enrofloxacin and its active metabolite ciprofloxacin after intravenous injection to cats with reduced kidney function
Source: J Vet Intern Med. 2023 Sep 20;37(6):2230–40. doi: 10.1111/jvim.16866 (PMC10658592; doi:10.1111/jvim.16866)
Supplement: Supplementary file 2 — Table S2. Results of 300 bootstrap simulations and original model estimates. [file JVIM-37-2230-s003.pdf]

Supplemental Table 2. Results of 300 bootstrap simulations and original model estimates.

Parameter	Bootstrap Mean Observation	Model Estimate	Value	Model Std Err	Bootstrap CV%	Model CV%
θ V_d1	4875.4	4793.26	mL/kg	327.84	9.56	6.84
V_d2	2.1×10^5	2222.57	mL/kg	1123.14	1677.84	50.53
θ Cl1	236.7	234.82	mL/kg/hr	45.86	209.2	19.53
Cl2	254.87	190.65	mL/kg/hr	53.41	400.62	28.02
dV_d1weight	1.29	0.97		0.22	29.02	22.72
dCl1weight	1.93	1.33		0.45	59.52	33.88

θ V_d1 is the theta (typical value) for quinolone volume of distribution of the first compartment; V_d2 is the volume of distribution of quinolone within the second compartment; θ Cl1 is the theta for quinolone clearance from the first compartment; Cl2 is the clearance of quinolone from the second compartment; dV_d1weight is the effect of the covariate weight on the volume of distribution of the first compartment; dCl1weight is the effect of the covariate weight on the quinolone clearance of the first compartment.
