# Supplementary figures and images for: Population pharmacokinetic analysis of enrofloxacin and its active metabolite ciprofloxacin after intravenous injection to cats with reduced kidney function
Source: J Vet Intern Med. 2023 Sep 20;37(6):2230–40. doi: 10.1111/jvim.16866 (PMC10658592; doi:10.1111/jvim.16866)

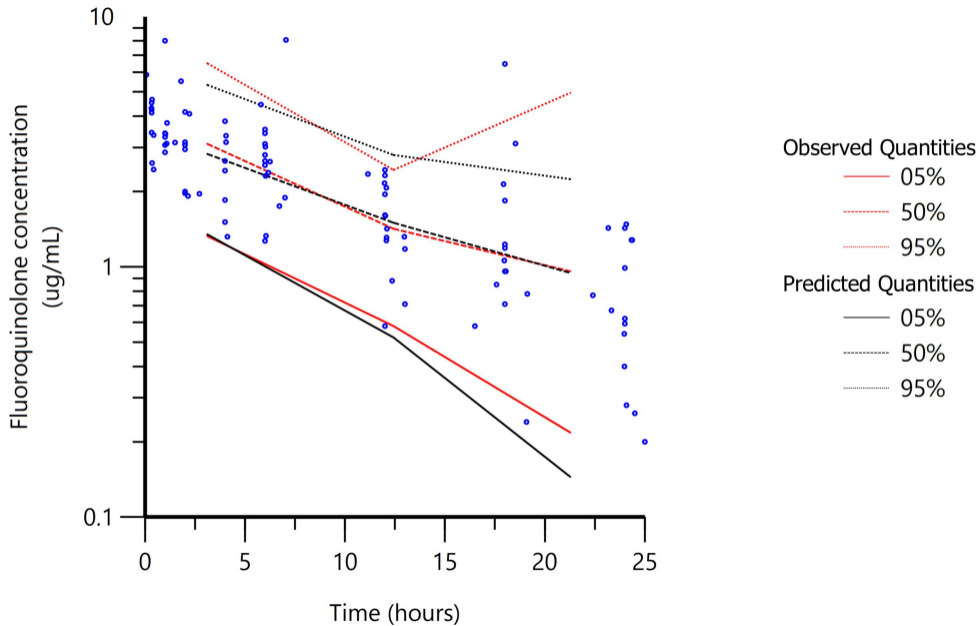

Supplement: Supplementary file 3 — Figure S1. Visual check of predicted and observed total fluoroquinolone concentrations vs time. [file JVIM-37-2230-s001.pdf]

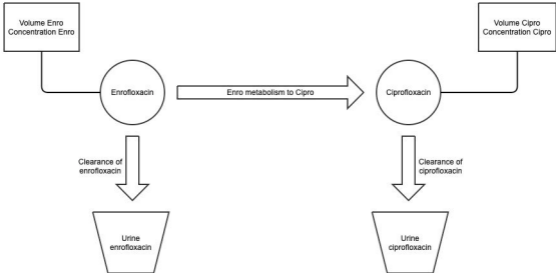

Supplement: Supplementary file 4 — Figure S2. Graphical model of enrofloxacin metabolism to ciprofloxacin and their respective clearances. [file JVIM-37-2230-s002.pdf]
